# Aspects cliniques de la dengue dans la région des Savanes au Togo en 2023

**DOI:** 10.48327/mtsi.v5i4.2025.788

**Published:** 2025-12-16

**Authors:** Lidaw Déassoua BAWE, Awèréou KOTOSSO, Komivi Atsu KPEGBA, Mawaba HILIM, Dondah KOZON, Bawoubadi ABALTOU, Akouda Akessiwe PATASSI, Majesté Ihou WATÉBA

**Affiliations:** 1 Service des maladies infectieuses et tropicales, Centre hospitalier universitaire Sylvanus Olympio, Lomé, Togo; 2 Centre hospitalier des armées de Lomé, Togo; 3 Centre hospitalier régional de Dapaong, Togo; 4 Direction préfectorale de la santé de Tône, Togo; 5 Direction préfectorale de la santé de Cinkassé, Togo; 6 Centre médical des armées du 1^er^ bataillon d’intervention rapide, Togo

**Keywords:** Épidémie, Dengue, Région des Savanes, Togo, Afrique subsaharienne, Epidemic, Dengue, Savanes Region, Togo, Sub-Saharan Africa

## Abstract

**Introduction:**

La région des Savanes au Togo a connu en 2023 la première grande épidémie de dengue du pays. Lobjectif de cette étude est de décrire les caractéristiques épidémiologique, clinique et évolutive des cas confirmés de dengue au cours de cette épidémie.

**Patients et méthode:**

Il s’est agi d’une étude rétrospective sur les cas de dengue confirmés lors de l’épidémie dans la région sanitaire des Savanes, du 15 septembre 2023 au 29 février 2024.

**Résultats:**

Durant l’épidémie, 57 cas suspects de dengue ont été rapportés dont 27 cas confirmés. La moyenne d’âge des patients était de 32 ans (10-65). Les signes cliniques étaient dominés par le syndrome algique, la fièvre (n = 19), l’asthénie (n = 17), l’anorexie (n = 12) et les vomissements (n = 11). La répartition des formes cliniques de dengue dénombrait 7 cas de dengue classique sans signe d’alerte, 15 cas de dengue avec signe d’alerte et 5 cas de dengue sévère. Les paramètres hématologiques montraient une augmentation du nombre de globules blancs surtout dans les formes sévères de dengue. Sur le plan biochimique, on notait une augmentation des paramètres rénaux et hépatiques dans la dengue avec signe d’alerte et la dengue sévère. Deux décès de dengue sévère ont été enregistrés, soit un taux de létalité d’un peu plus de 7%.

**Conclusion:**

La présente étude rapporte la première grande épidémie de dengue que le Togo a connue dans sa région septentrionale. Elle a permis de noter une proportion non négligeable de cas de dengue avec formes intermédiaires (signes d’alerte) et sévères.

## Abstract (en)

**Introduction:**

In 2023, the Savanes region of Togo experienced the country’s first major dengue epidemic. This study aims to describe the epidemiological, clinical and evolutionary characteristics of confirmed dengue cases during this epidemic.

Patients and methods. This retrospective study examined confirmed dengue cases in the Savanes health region from 15 September 2023 to 29 February 2024.

Results. During the epidemic, 57 suspected cases of dengue were reported, of which 27 were confirmed. The average patient age was 32 years (range 10-65 years). The main clinical signs were a pain syndrome (n = 19), fever (n = 19), asthenia (n = 17), anorexia (n = 12) and vomiting (n = 11). The distribution of dengue clinical forms included seven cases of classic dengue without warning signs, 15 cases of dengue with warning signs, and five cases of severe dengue. Haematological parameters showed an increase in white blood cell count, particularly in severe dengue cases. Biochemically, renal and hepatic parameters increased in cases of dengue with warning signs and severe dengue. Two deaths from severe dengue were recorded, representing a case fatality rate of just over 7%.

**Conclusion:**

This study reports the first major dengue epidemic in northern Togo. A significant proportion of cases were found to be intermediate (warning signs) or severe.

## Introduction

La dengue est une arbovirose endémique dans la plupart des pays tropicaux et subtropicaux transmise par des moustiques diurnes du genre *Aedes* sous-genre *Stegomyia.* Le virus a été isolé des espèces *Aedes furcifer* et *Aedes luteocephalus* en zone sylvatique, et des espèces *Aedes aegypti* et *Aedes albopictus* en zone urbaine [3].

Les virus de la dengue (sérotypes DENV1 à 4) sont les arbovirus pathogènes humains les plus importants. En milieu urbain, les virus (DENV 1, DENV2, DENV3, DENV 4) sont transmis entre hôtes humains par les moustiques péridomestiques *Ae. aegypti* et *Ae albopictus.* Des études sur l’écologie du virus de la dengue en milieu sylvatique d’Afrique de l’Ouest et de Malaisie ont identifié des cycles de transmission impliquant des primates non humains pour les virus DENV 1, DENV2 et DENV4).

Environ 50 à 100 millions de cas de dengue sont rapportés chaque année dans le monde par l’Organisation mondiale de la santé [2].

La résolution WHA55. 17 de l’Assemblée mondiale de la santé de 2002 a appelé l’OMS et ses États-membres à s’engager davantage dans la lutte contre la dengue [18]. La résolution WHA58. 3 de l’Assemblée mondiale de la santé de 2005 sur la révision du Règlement sanitaire international (RSI) revêt une importance particulière. Elle inclut la dengue comme exemple de maladie pouvant constituer une urgence de santé publique de portée internationale avec des implications pour la sécurité sanitaire, en raison des perturbations et de la propagation rapide de l’épidémie au-delà des frontières nationales [19].

L’Afrique de l’Ouest est une zone endémique de dengue du fait de la croissance rapide des zones urbaines sans assainissement adéquat, ce qui crée des conditions favorables à la prolifération du vecteur *Aedes aegypti* [1,13]. La circulation du virus chez les populations humaines a été décrite pour la première fois dans les années 1960 au Nigeria et depuis, plusieurs pays d’Afrique ont signalé des cas sporadiques ou des flambées épidémiques associées à ce virus [13]. Selon l’OMS, « la dengue continue d’être sous-déclarée en Afrique en raison d’un manque de sensibilisation des prestataires de soins de santé, de la présence d’autres maladies fébriles (notamment le paludisme)… » [15].

Au Togo, l’épidémiologie de la dengue est mal connue, mais des études ont montré la circulation du virus chez les humains dans le pays, avec, par moment, des poussées épidémiques. La dernière occurrence de dengue connue au Togo remonte à 2017 avec 12 cas confirmés à Lomé sans décès. La récente épidémie en 2023 a eu lieu dans la région des Savanes qui fait frontière avec le Burkina Faso où la circulation du virus de la dengue est active (données non publiées). Toujours en 2017, au Centre hospitalier universitaire Sylvanus Olympio de Lomé, une étude sur la prévalence de la dengue chez les patients présentant un syndrome fébrile a montré qu’en réalité, il y avait plus de cas de dengue (17%) que de paludisme (10,2%) [17]. L’objectif de cette étude est de décrire les caractéristiques épidémiologiques, cliniques, paracliniques et évolutives de l’épidémie qu’a connue le Togo dans la région des Savanes en 2023.

## Patients et méthode

Il s’agissait d’une étude rétrospective sur les cas de dengue enregistrés lors de l’épidémie au Togo notamment dans la région sanitaire des Savanes. Cette région couvre une superficie de 8 470 km^2^ avec une population estimée à 1 169 821 habitants en 2022. Elle fait frontière avec le Burkina Faso au nord, le Bénin à l’est et le Ghana à l’ouest, des pays ayant tous connu des épidémies de dengue ou au moins des cas autochtones [13]. En août 2023, la situation épidémiologique de la maladie a pris une tournure alarmante au Burkina-Faso avec une explosion des cas suspects et une série de décès [8].

À partir de ce moment, un niveau d’alerte élevé a été maintenu au Togo pour une détection précoce et une notification rapide des cas, le renforcement de la surveillance aux points d’entrée, l’organisation de la prise en charge d’éventuels cas et la sensibilisation des populations [21]. Le système de surveillance du Togo a commencé l’enregistrement des données à partir du 15 septembre 2023 et, selon la liste linéaire, le dernier cas suspect a été notifié le 10 janvier 2024. À la date du 14 janvier 2024, la Division de la surveillance intégrée des urgences sanitaires et de la riposte du ministère de la santé et de l’hygiène publique du Togo a déclaré qu’aucun cas confirmé de dengue n’avait été enregistré [8] et ce pendant 6 semaines. La période considérée de cette épidémie allait ainsi du 15 septembre 2023 au 29 février 2024.

**Figure 1:**
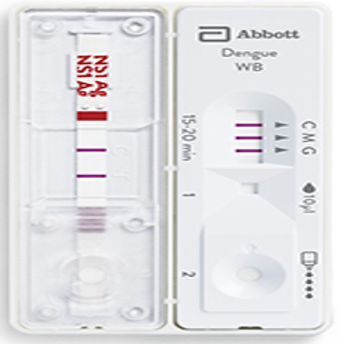
Test rapide Bioline™ Dengue Duo

Le Centre hospitalier régional (CHR) de Dapaong, chef-lieu de la région des Savanes, a servi de centre de prise en charge des cas de dengue. Le cas suspect de dengue était défini comme toute personne présentant une maladie fébrile (>39°C) aiguë d’une durée comprise entre deux et sept jours, et s’accompagnant d’au moins deux des symptômes suivants: céphalées, douleurs rétroorbitales, myalgies, arthralgies, éruptions cutanées, manifestations hémorragiques, syndrome de choc. Le cas confirmé a été défini comme tout cas suspect confirmé par le laboratoire [12]. La confirmation biologique a été faite à partir du test rapide Bioline^TM^ Dengue Duo (Société Abbott, Santa Clara, Californie, USA), un test immunochromatographique *in vitro* conçu pour détecter à la fois l’antigène NS1 du virus de la dengue et les anticorps IgM/IgG contre le virus dans le sérum, le plasma ou le sang total humain. Le test rapide Bioline^TM^ Dengue Duo contient deux dispositifs de test (côté gauche: test Dengue NS1 Ag, côté droit: test Dengue IgG/IgM) (Fig. 1).

**Figure 2:**
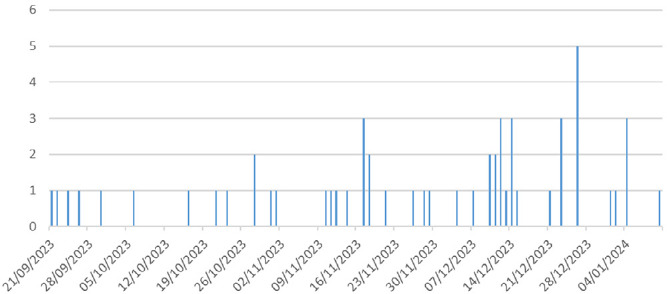
Évolution hebdomadaire des cas suspects de dengue dans la région des Savanes entre le 21 septembre 2023 et le 10 janvier 2024

Le résultat est obtenu en 15-20 minutes. La dengue était confirmée en cas de détection de l’antigène NS1 du virus et/ou des anticorps IgM. Compte tenu du coût de la détection du génome viral par la réaction de polymérase en chaîne, la biologie moléculaire (qRT-PCR) n’a été pratiquée que pour les premiers cas de dengue suspects chez lesquels le test rapide était positif selon la définition de cas. Les données ont été extraites d’une base EXCEL de la liste linéaire de surveillance où tous les cas suspects et confirmés étaient enregistrés par le Point focal de la Surveillance intégrée des maladies et de la riposte (SIMR) de la région des Savanes. La Figure 2 montre l’évolution hebdomadaire des cas suspects.

Les paramètres étudiés étaient le sexe, l’âge, la notion de voyage dans les pays voisins, les données cliniques, les données des résultats du test rapide Bioline™ Dengue Duo, les données paracliniques (hématologiques et biochimiques) et les données évolutives (complications, durée d’hospitalisation et issue).

Trois formes ou présentations cliniques de la dengue ont été définies:

- la dengue classique, sans signe d’alerte, est marquée par des signes courants: fièvre, rougeur du visage, érythème, douleurs corporelles généralisées, myalgie, arthralgie, douleurs oculaires rétro-orbitaires, photophobie, exanthème rubéoliforme, céphalées, anorexie, nausées et vomissements [19];
- la dengue avec signes d’alerte, est caractérisée par les signes de la dengue classique associés à des signes d’alerte tels que douleur ou sensibilité abdominale, vomissements persistants, épanchement liquidien/accumulation clinique de liquide (pleural, péritonéal, péricardique…), saignement des muqueuses, léthargie, agitation, hépatomégalie, augmentation de l’hématocrite et diminution du nombre de plaquettes [19];
- la dengue grave est caractérisée par:
  - une fuite plasmatique grave entraînant un état de choc et/ou un épanchement liquidien avec détresse respiratoire;
  - une hémorragie sévère;
  - une défaillance grave des organes: cytolyse hépatique avec aspartate aminotransférase (ASAT) ou alanine aminotransférase (ALAT) ≥ 1 000 UI/l; altération de la conscience; défaillance cardio-vasculaire, rénale…) [19].

Une autorisation a été obtenue auprès de la Division de la surveillance intégrée des urgences sanitaires et de la riposte au ministère de la santé et de l’hygiène publique du Togo, ce qui a permis d’avoir accès à la liste linéaire élaborée par le Point focal de la surveillance de la région des Savanes. L’anonymat et la confidentialité des patients ont été respectés.

## Résultats

Durant l’épidémie, les données de la surveillance ont colligé 57 cas suspects de dengue répondant à la définition de cas, parmi lesquels 27 cas étaient confirmés au CHR de Dapaong, soit une fréquence de 47%. Les taux d’incidence et d’attaque étaient respectivement de 2,3 pour 100 000 habitants et 0,0023%. Il n’y avait aucune donnée sur les cas communautaires de dengue qui n’ont pas été vus à l’hôpital. Seuls les patients fébriles et répondant à la définition de cas suspects ont été enregistrés.

### Caractéristiques socio-démographiques et notions de voyages

#### Cas suspects

Leur moyenne d’âge était de 27,16 ans avec des extrêmes de 3 ans et 63 ans. Ils provenaient du milieu urbain (n = 18) et rural (n = 12). On notait 11 enfants dont l’âge moyen était de 8,72 ans [3 ans-17 ans]. Les cultivateurs (n = 7) et les ménagères (n = 6) étaient majoritaires. Deux patients avaient un antécédent de voyage au Burkina Faso et au Mali.

#### Cas confirmés de dengue

Seize patients (16/27) étaient de sexe masculin. La moyenne d’âge des patients était 32,29 ans avec des extrêmes allant de 10 ans à 65 ans. Neuf patients provenaient des pays frontaliers avec la région des Savanes dont sept patients venant du Burkina-Faso et deux patients respectivement du Ghana et de la Côte d’Ivoire. La majorité vivait en milieu urbain (n=25); les professions représentées étaient disparates allant des fonctionnaires (n = 7) dont 4 personnels de santé, des ménagères (n = 7) et plusieurs professions libérales. On dénombrait deux enfants de 10 et 17 ans.

#### Caractéristiques cliniques et paracliniques

Les signes cliniques classiques de la dengue présentés par les patients étaient dominés par le syndrome algique (22 cas de céphalées, 12 cas de douleurs musculaires), la fièvre (n = 19), l’asthénie (n = 17) dont 15 cas de fatigue intense, l’anorexie (n = 12) et les vomissements (n = 11) (Tableau I). La répartition des formes cliniques de dengue dénombrait 11 cas de dengue classique, 9 cas de dengue avec signe d’alerte et 7 cas de dengue sévère.

**Tableau I:** Caractéristiques cliniques des cas confirmés de dengue au cours de l’épidémie dans la région des Savanes au Togo en 2023 (n = 27)

Signes cliniques	Effectif (n)
Céphalées	22
Fièvre	19
Asthénie	17
Anorexie	12
Douleurs musculaires	12
Vomissements	11
Toux	7
Hématémèse	5
Dysphagie, diarrhée, épistaxis (***)	3
Difficultés respiratoires, douleurs épigastriques, érup-tion cutanée, vertiges, méléna, hématurie (**)	2
Otalgie, douleurs abdominales, constipation, prurit, hémolyse, hémoglobinurie (*)	1

* 1 cas de chaque signe

** 2 cas de chaque signe

*** 3 cas de chaque signe

Les signes de dengue sévère étaient une hématémèse dans six cas, une insuffisance rénale dans quatre cas, une cytolyse hépatique dans quatre cas, une hématurie dans deux cas, une hémolyse et une encéphalopathie hépatique avec altération de la conscience dans un cas.

Dans la dengue avec signe d’alerte, on notait principalement des vomissements persistants (n = 7), une épistaxis (n = 3), des douleurs abdominales et épigastriques (n = 2) et un cas d’hémoglobinurie. Chez les cas suspects de dengue (n = 30), le Tableau II montre que la présentation clinique était en majorité marquée par la fièvre (n = 25), les céphalées (n=22) et les éruptions cutanées (n = 12). Les paramètres hématologiques montraient une augmentation du nombre de globules blancs dans les formes sévères de dengue. Sur le plan biochimique, on notait une augmentation des paramètres rénaux (urée et créatinine) et hépatiques (alanine aminotransférase et aspartate aminotransférase) dans la dengue avec signe d’alerte et dans la dengue sévère (Tableau III). La détection de l’antigène NS1 du virus était positive dans 12 cas. Les immunoglobulines IgM et IgG étaient respectivement positives dans 26 et 17 cas. La détection de l’antigène NS1 et des immunoglobulines (Ig M et Ig G) en fonction du tableau clinique de dengue est présentée dans le Tableau IV.

**Tableau II:** Caractéristiques cliniques des cas suspects de dengue au cours de l’épidémie dans la région des Savanes au Togo en 2023 (n = 30)

Signes cliniques	Effectif (n)
Fièvre	25
Céphalées	22
Éruptions cutanées	12
Douleurs musculaires	9
Fatigue intense	7
Vomissement	6
Toux	6
Anorexie	5
Courbatures	3
Conjonctivite, colique abdominale, prurit (*)	2
Diarrhée, écoulement nasal, otalgie (**)	**1**

* 2 cas dans chaque signe;

** 1 cas dans chaque signe

**Tableau III:** Données hématologiques et biochimiques des patients atteints de dengue au cours de l’épidémie dans la région des Savanes au Togo en 2023 (n = 27)

	Dengue classique (n = 11)	Dengue avec signe d’alerte (n = 9)	Dengue sévère (n = 7)
Hématologie			
Globules blancs (cellules/mm^3^)	4 403,28 [2 000-10 000]	7 994,66 [1 800-14 920]	36 080 [15 900-59 900]
Plaquettes (cellules/mm^3^)	188 090,90 [140 000-308 000]	115 444,44 [75 000-205 000]	84 142,85 [48 000-109 000]
Taux d’hémoglobine (g/dl)	10,82 [9,7-13]	11,72 [6,2-15,2]	9,9 [8-12]
Biochimie			
Urémie (g/l)	0,25 [0,1-0,45]	0,496 [0,21-0,52]	1,6 [1-2,04]
Créatininémie (mg/l)	10,42 [7-14]	14,86 [8-19]	85,6 [36-125]
ALAT (UI/l)	48,14 [30-74]	166,33 [49-302]	1 030,2 [366-1 895]
ASAT (Ul/l)	70,14 [37-109]	236,8 [94-498]	1 143 [328-1 825]

ALAT: alanine aminotransférase; ASAT: aspartate aminotransférase

**Tableau IV:** Répartition de la détection de l’antigène NS1, des immunoglobulines M et G selon la forme clinique de dengue chez les patients au cours de l’épidémie dans la région des Savanes au Togo en 2023 (n = 27)

	Dengue classique (n = 11)	Dengue avec signe d’alerte (n = 9)	Dengue sévère (n = 7)
	Positif	Négatif	Positif	Négatif	Positif	Négatif
Antigène NS1	3	8	5	4	4	3
Ig M	11	0	8	1	7	0
Ig G	7	4	4	5	6	1

#### Données *thérapeutiques*

Un traitement symptomatique a été appliqué à tous les patients sous diverses formes. Le syndrome algique et la fièvre ont été gérés par du paracétamol (n = 25) et du métamizole (n = 20). Douze patients ont bénéficié d’un traitement antiémétique à base de métopimazine. Des perfusions de vitamine C ont été administrées à 12 patients, des hémostatiques (étamsylate) prescrits chez 7 patients. Des transfusions (culot globulaire et concentré plaquettaire) ont été nécessaires chez 6 patients.

#### Données *évolutives*

Chez un patient présentant une dengue sévère dans la forme hémorragique, l’évolution a été marquée par:

- une hépato-néphrite faite de cytolyse hépatique et hémolyse avec ictère, insuffisance rénale aiguë avec oligo-anurie et urines porto (hémolyse);
- une détresse respiratoire avec désaturation sous ventilation non invasive (saturation en oxygène à 86%);
- un sepsis.

Secondairement, son évolution a été favorable après son évacuation au centre de prise en charge des maladies à potentiel épidémique à Lomé. Par ailleurs, on a dénombré 2 décès, soit une létalité d’un peu plus de 7%. Le premier cas de décès était survenu chez un patient de 25 ans sans pathologie associée dans un tableau d’hémorragie digestive à type d’hématémèse, de défaillance multiviscérale (insuffisance rénale cytolyse hépatique) et de sepsis sévère. Le second décès, une patiente de 65 ans sans antécédent pathologique, dans un tableau d’insuffisance rénale, de cytolyse hépatique avec encéphalopathie hépatique.

La durée moyenne d’hospitalisation était de 4,44 jours avec des extrêmes de 1 et 8 jours.

## Discussion

Le Togo, pays frontalier avec le Burkina Faso qui connaît fréquemment des épidémies de dengue, vient de connaître sa première grande épidémie dans la région des Savanes, bien que des études aient déjà montré la circulation du virus de la dengue dans le pays comme dans les régions de la Kara [24] et du Grand Lomé [17]. Les données de la surveillance ont permis de colliger 57 cas suspects dont 27 cas confirmés avec 2 décès dénombrés, soit une létalité de 7%. En réalité, ces chiffres seraient revus à la hausse si la notification était efficace et les tests de diagnostic disponibles. Bien que la maladie soit présente au Togo et que le pays soit vraiment à risque de dengue du fait de sa proximité avec le Burkina Faso, la préparation et la réponse à une éventuelle épidémie n’ont pas vraiment suivi. L’absence de disponibilité des tests rapides sur place a conduit au convoyage des prélèvements des cas suspects au laboratoire national de santé publique (Institut national d’hygiène) situé à Lomé à 600 km de la région des Savanes au début de l’épidémie. Cette difficulté de l’accessibilité aux kits de test de diagnostic rapide de la dengue au laboratoire représente déjà un biais de recrutement. La liste serait plus importante si le diagnostic avait été possible sur place au début de l’épidémie.

Le risque de survenue d’une épidémie de dengue reste élevé au Togo puisque c’est une maladie émergente liée à la prolifération du vecteur *Aedes* due à la multiplication des gites larvaires. Les programmes de lutte anti-vectorielle connaissent des défaillances et le changement climatique est à l’œuvre [22]. Cette prolifération du vecteur s’explique par les puisards débordant dans les quartiers, un système de drainage des eaux défectueux ou inexistant entraînant la stagnation de l’eau, un terrain propice à la reproduction des moustiques. La démographie avec l’urbanisation incontrôlée et les lacunes des politiques sanitaires dans la lutte contre les moustiques contribuent également à cette prolifération [8]. Le niveau socioéconomique du pays participe à la vulnérabilité des populations face à ces maladies: non-accès à l’eau potable, points d’eaux usées propices au développement et à la pullulation des moustiques. De plus, la déforestation, certaines pratiques agricoles, l’utilisation non contrôlée des insecticides sont d’autres facteurs qui favorisent la transmission des agents pathogènes par les vecteurs.

*Aedes aegypti* est hautement anthropophile. En raison de leur forte domestication, les femelles pondent leurs œufs dans des gîtes artificiels tels les réservoirs d’eau, les pots de fleurs, les pneus usagés, les gouttières. Il a été également démontré que les œufs pouvaient résister à la dessiccation pendant au moins un an [5].

Une collecte d’information réalisée en 2016 par l’OMS et basée sur diverses études et enquêtes dans la région africaine, a observé une recrudescence du vecteur *Ae. aegypti* dans toute l’Afrique y compris certains pays de l’Afrique du Nord [6]. Ce moustique est aussi vecteur d’autres maladies virales comme la fièvre jaune, la fièvre à virus Zika, ou encore la maladie à virus chikungunya. Les risques d’épidémie majeure par la transmission de la maladie sont réels et constituent une véritable menace pour le continent et la santé des populations. D’autres vecteurs de la maladie non natifs d’Afrique, comme *Ae. albopictus,* plus communément connu sous le nom de moustiquetigre se sont introduits dans certaines régions d’Afrique [6], notamment en Afrique de l’Ouest (Nigéria, Bénin), en Afrique centrale et de l’Est (Gabon et Seychelles) [3,23].

Cette recrudescence d’insectes vecteurs de maladies infectieuses est attribuée en partie à l’augmentation des échanges et du tourisme internationaux, mais surtout au changement climatique. D’après certaines études réalisées à l’échelle globale, le changement climatique aurait pour effet d’accroître les zones où le climat est plus propice à la multiplication de ces insectes et des épidémies qui leur sont associées [6]. Le rapport 1. 5 sur le climat et la santé, publié en octobre 2019 par *l’Intergovernmental Panel on Climate Change* (IPCC), l’annonce clairement: « Les preuves sont de plus en plus irréfutables que les variations météorologiques associées au changement climatique modifient l’étendue géographique, les saisonnalités ainsi que l’intensité de la transmission des maladies infectieuses ». En d’autres termes, les vecteurs *Aedes* pourraient se répandre bien au-delà de leur zone actuelle d’ici à 2030, de même que le moustique Anophèle, vecteur du paludisme pourrait changer de zone géographique en fonction du réchauffement climatique. Une poussée de cette maladie, la plus fatale pour l’Afrique, a d’ailleurs été notée récemment dans beaucoup de pays du continent [6].

La distribution géographique actuelle du moustique tigre *Ae. albopictus* englobe des régions du monde qui présentent une similarité climatique avec Chiba au Japon, le berceau d’origine de ce moustique [6].

Cette épidémie est survenue au moment où plusieurs pays voisins tels que le Burkina Faso, le Ghana, le Bénin, la Côte d’Ivoire et le Mali étaient aussi en épidémie [16].

Le Burkina Faso est l’un des pays limitrophes du Togo et directement frontalier à la région des Savanes. Depuis la première épidémie de dengue au Burkina Faso en 1925, plusieurs autres ont suivi depuis les années 2000, dont celles de 2013,2016 et 2017 [10,14].

Au 19 décembre 2023, un total de 171 991 cas de dengue, dont 70 223 cas confirmés et probables et 753 décès (létalité: 0,4%) ont été signalés au Burkina Faso, au Cap-Vert, au Tchad, en Côte d’Ivoire, en Éthiopie, en Guinée, au Mali, à Maurice, à São Tomé-et-Principe, au Sénégal, au Nigéria, au Ghana, au Bénin et au Togo [19]. Le Burkina Faso représentait 85% du total des cas (n = 146 878) et 91% des décès (n = 688) [16].

### Caractéristiques sociodémographiques

La tendance observée au cours de l’épidémie qui montrait que la dengue touchait une population jeune, a été constatée dans plusieurs épidémies en Afrique de l’Ouest tels que le Burkina-Faso [1,4, 7,14].

### Données cliniques

Les données résultant de plusieurs études sur la dengue rapportent une prédominance de cas de dengue classique avec des signes de syndrome pseudo-grippal dominés par le syndrome algique comme dans notre série, avec céphalées, myalgies, douleurs abdominales, douleurs rétro-orbitaires, cervicalgies [4,11].

Le syndrome hémorragique était essentiellement marqué dans notre série par l’hématémèse, l’épistaxis, le méléna et l’hématurie. Au Burkina Faso entre 2013 et 2017, le rapport des cas de dengue avait notifié les gingivorragies, hématuries, hématémèses, métrorragies et épistaxis [4]. Au Sénégal lors d’une épidémie en 2009, l’épistaxis et le méléna venaient au premier rang [7].

### Données paracliniques

L’analyse des données hématologiques et biochimiques de la série montre, par rapport à la classification des différentes présentations cliniques, que plus le tableau évolue, plus on passe de la leucopénie (dengue classique) à une hyperleucocytose (dengue sévère).

Nous avons également observé une augmentation des transaminases associée à la dengue surtout dans la fraction des ASAT, comme le prouve une étude au Singapour où sur une série de 690 patients atteint de dengue confirmée on en notait 595 (soit 86%) qui avaient une élévation de l’ASAT quelle que soit la présentation clinique. Plus la dengue est sévère, plus les ASAT sont élevées [9]. Ceci est tout à fait conforme aux critères de classification de l’OMS [20].

Au vu du risque que représente la dengue au Togo, le pays a mis en place plusieurs mesures de renforcement de la surveillance aux frontières. La surveillance transfrontalière de la dengue a été intensifiée par plusieurs actions parmi lesquelles le *briefing* des agents sanitaires aux points d’entrée, la sensibilisation des voyageurs et des populations locales sur les risques sanitaires, le *briefing* des agents de santé des zones à risque sur la détection précoce des cas et leur prise en charge, ainsi que l’acquisition et la mise à disposition des intrants nécessaires à la prise en charge. À l’échelle nationale, un plan d’action a été mis en place, englobant l’organisation de la prise en charge des cas dans les établissements de santé, le renforcement de la détection précoce grâce à des confirmations en laboratoire, et la sensibilisation de la population aux mesures préventives contre la dengue [8].

## Conclusion

La présente étude rapporte les caractéristiques épidémiologiques de la première grande épidémie de dengue que le Togo ait connue dans la région septentrionale frontalière avec le Burkina Faso, pays endémique de dengue et où des épidémies sont fréquentes. Elle a permis de noter une proportion non négligeable de cas de dengue avec signe d’alerte. Toutefois, les sérotypes du virus n’ont pu être identifiés. Des études ultérieures doivent être menées pour connaître les sérotypes circulants et ceux associés aux cas de dengue sévère dans cette région du Togo et dans tout le pays en général.

La proximité de la région des Savanes avec le Burkina Faso où la circulation du virus est plus active fait de cette région une zone à risque élevé de dengue et, par ricochet, le Togo entier. Il apparaît que la région Nord-Togo-Burkina Faso est devenue endémo-épidémique pour la dengue. Ce constat doit conduire à l’instauration d’une surveillance épidémiologique coordonnée des arboviroses dans cette région, afin de préciser leur incidence et les virus en cause, et de les distinguer du paludisme.

## Contribution des auteurs

Kpegba KA, Hilim M et Kozon D ont supervisé régulièrement la mise à jour de la liste linéaire des cas de dengue dans la région des Savanes.

Bawe LD, Kotosso A et Abaltou B ont rédigé le protocole de l’étude.

Bawe LD, Kotosso A et Abaltou B ont analysé les résultats et rédigé la première version du manuscrit.

Patassi AA, Watéba MI ont validé les résultats et corrigé la première version du manuscrit.

## Déclaration de lien d’intérêt

Les auteurs ne déclarent aucun lien d’intérêt.
