# Supplementary material for: Clinical aspects of dengue fever in the Savanna region of Togo in 2023
Source: Med Trop Sante Int. 2025 Dec 16;5(4):mtsi.v5i4.2025.788. [Article in French] doi: 10.48327/mtsi.v5i4.2025.788 (PMC12957991; doi:10.48327/mtsi.v5i4.2025.788)
Supplement: Related texts

# Research article: Clinical aspects of dengue fever in the Savanna region of Togo in 2023

## Introduction

Dengue fever is an arbovirus disease that is endemic in most tropical and subtropical countries. It is transmitted by daytime mosquitoes belonging the *Stegomyia* subgenus of the *Aedes* species. The virus has been isolated from *Ae. furcifer* and *Ae. luteocephalus* in sylvatic areas and from *Ae. aegypti* and *Ae. albopictus* in urban areas [3].

Dengue viruses (DENV1 to DENV4) are the most significant human-pathogenic arboviruses. In urban areas, these viruses (DENV1, DENV2, DENV3, DENV 4) are transmitted between human hosts by the peridomestic mosquitoes *Ae. aegypti* and *Ae. albopictus.* Studies on the ecology of the dengue virus in forest environments in West Africa and Malaysia have identified transmission cycles involving non-human primates for DENV1, DENV2 and DENV4.

The World Health Organization (WHO) reports that approximately 50 to 100 million cases of dengue are reported each year worldwide [2].

Resolution WHA55.17 of the 2002 World Health Assembly called on the WHO and its Member States to increase their commitment to fighting dengue [18]. Of particular importance is Resolution WHA58.3 of the 2005 World Health Assembly on the revision of the International Health Regulations (IHR). It includes dengue as an example of a disease that may constitute a public health emergency of international concern, with implications for health security due to disruption and the rapid spread of epidemics beyond national borders [19].

West Africa is a dengue endemic area due to rapid urbanisation and uncontrolled population growth, which creates favourable conditions for the proliferation of the *Ae. aegypti* vector [1,13]. The first description of the virus circulating in human populations was in Nigeria in the 1960s, and since then several other African countries have reported sporadic cases or outbreaks associated with the virus [13]. According to the WHO, “Dengue continues to be underreported in Africa due to a lack of awareness among healthcare providers and the presence of other febrile illnesses, particularly malaria…” [15].

The epidemiology of dengue in Togo is poorly understood, but studies have shown that the virus circulates among the population, occasionally resulting in outbreaks. The last known occurrence of dengue fever in Togo dates back to 2017, with 12 confirmed cases in Lomé and no fatalities. The most recent epidemic occurred in the Savanes region in 2023, which borders Burkina Faso, where the dengue virus is actively circulating (unpublished data). In 2017, a study on the prevalence of dengue among patients with fever syndrome at the Sylvanus Olympio University Hospital Centre in Lomé showed that there were actually more cases of dengue (17%) than malaria (10.2%) [17]. This study aims to describe the epidemiological, clinical, paraclinical and evolutionary characteristics of the 2023 epidemic in Togo’s Savanes region.

## Patients and methods

This was a retrospective study of dengue cases recorded during the epidemic in Togo, particularly in the Savanes health region. This region covers an area of 8,470 km^2^ and had an estimated population of 1,169,821 in 2022. It borders Burkina Faso to the north, Benin to the east and Ghana to the west, all of which have experienced dengue epidemics or indigenous cases [13]. In August 2023, the epidemiological situation in Burkina Faso took an alarming turn with a surge in suspected cases and a series of deaths [8].

From that point on, Togo maintained a high level of alert for the early detection and rapid notification of cases. This involved strengthening surveillance at points of entry, organising the management of possible cases and raising awareness among the population [21]. Togo’s surveillance system began recording data on 15 September 2023, and according to the linear list, the last suspected case was reported on 10 January 2024. As of 14 January 2024, Togo’s Ministry of Health and Public Hygiene’s Integrated Health Emergency Surveillance and Response Division reported that no confirmed cases of dengue fever had been recorded for six weeks [8]. The period under consideration for this epidemic was there-fore from 15 September 2023 to 29 February 2024. The Regional Hospital Centre (CHR) in Dapaong, the capital of the Savanes region, served as the centre for managing dengue cases. A suspected case of dengue fever was defined as a person presenting with an acute fever (>39°C) lasting between two and seven days, accompanied by at least two of the following symptoms: headache, retro-orbital pain, myalgia, arthralgia, rash, haemorrhagic manifestations or shock syndrome. A confirmed case was defined as any suspected case that was confirmed by laboratory testing [12]. Biological confirmation was performed using the Bioline^TM^ Dengue Duo rapid test (Abbott, Santa Clara, California, USA). This is an in vitro immunochromatographic test designed to detect the NS1 antigen of the dengue virus, as well as IgM and IgG antibodies against the virus, in human serum, plasma or whole blood. The Bioline^TM^ Dengue Duo rapid test contains two test devices (Dengue NS1 Ag test on the left and Dengue IgG/IgM test on the right) (Fig. 1).

**Figure 1:** Bioline^TM^ Dengue Duo rapid test

Results are obtained within 15-20 minutes. Due to the cost of detecting the viral genome using the polymerase chain reaction, qRT-PCR was only performed on the first suspected dengue cases in which the rapid test was positive according to the case definition.

The data were extracted from an Excel database of the linear surveillance list, in which the Integrated Disease Surveillance and Response (SIMR) Focal Point for the Savanes region recorded all suspected and confirmed cases. Figure 2 shows the weekly evolution of suspected cases.

**Figure 2:** Weekly trends in suspected dengue cases in the Savannah region between September 21, 2023 and January 10, 2024

The studied parameters were gender, age, travel history to neighboring countries, clinical data, Bioline^TM^ Dengue Duo rapid test results, para-clinical data (hematological and biochemical), and outcome data (complications, length of hospitalization, and outcome).

Three clinical forms of dengue were defined:

- Classic dengue, without warning signs, is characterized by fever, facial flushing, erythema, generalized body aches, myalgia, arthralgia, retro-orbital eye pain, photophobia, a rubella-like rash, headache, anorexia, nausea, and vomiting [19].
- Dengue with warning signs, which is characterized by the signs of classic dengue associated with warning signs, such as abdominal pain or tenderness, persistent vomiting, fluid effusion/clinical fluid accumulation (pleural, peritoneal, pericardial, etc.), mucosal bleeding, lethargy, agitation, hepatomegaly, increased hematocrit, and decreased platelet count [19];
- Severe dengue, characterized by:
  -severe plasma leakage leading to shock and/or fluid effusion with respiratory distress;
  -severe hemorrhage;
  -severe organ failure (e.g., hepatic cytolysis with aspartate aminotransferase (AST) or alanine aminotransferase [ALT] >1,000 IU/L, altered consciousness, cardiovascular or renal failure, etc.) [19].

Authorization was obtained from the Integrated Health Emergency Surveillance and Response Division of the Ministry of Health and Public Hygiene of Togo, which provided access to the linear list compiled by the Savanes Region Surveillance Focal Point. Patient anonymity and confidentiality were respected.

## Results

During the epidemic, surveillance data revealed 57 suspected dengue cases that met the case definition. Of these cases, 27 were confirmed at the Dapaong Regional Hospital Center, representing a frequency of 47%. The incidence and attack rates were 2.3 per 100,000 people and 0.0023%, respectively. There were no data on community cases of dengue that were not seen at the hospital. Only patients with fever who met the case definition for suspected dengue cases were recorded.

### Socio-demographic characteristics and travel history

#### Suspected cases

The average age of patients was 27.16 years, ranging from 3 to 63 years of age. They came from urban (n = 18) and rural (n = 12) areas. Eleven children were included, with an average age of 8.72 years (range 3-17 years). The majority were farmers (n = 7) and housewives (n = 6). Two patients had a travel history to Burkina Faso and Mali.

#### Confirmed cases of dengue

Sixteen patients (16/27) were male. The average age of patients was 32.29 years, ranging from 10 to 65 years old. Nine patients came from countries bordering the Savanes region, including seven from Burkina Faso, two from Ghana, and two from Cote d’Ivoire. Most lived in urban areas (n = 25), and their professions ranged from civil servants (n = 7), including four health workers, to housewives (n=7) and other private professionals. There were also two children, aged 10 and 17.

#### Clinical and paraclinical characteristics

The patients presented with the classic clinical signs of dengue, which included pain syndrome (22 cases of headache and 12 cases of muscle pain), fever (19 cases), asthenia (17 cases), intense fatigue (15 cases), anorexia (12 cases), and vomiting (11 cases) (Table I)

**Table I:** Clinical characteristics of dengue confirmed cases during the outbreak in the Savanna region of Togo in 2023 (n = 27)

Clinical signs	Headcount (n)
Headaches	22
Fever	19
Anorexia	17
Asthenia	12
Muscle pain	12
Vomiting	11
Cough	7
Hematemesis	5
Dysphagia, diarrhea, epistaxis (***)	3
Respiratory difficulties, epigastric pain, rash, dizziness, melena, hematuria (**)	2
Earache, abdominal pain, constipation, pruritus, hemolysis, hemoglobinuria (*)	1

* 1 case of each sign

** 2 cases of each sign

*** 3 cases of each sign

The distribution of dengue clinical forms included 11 cases of classic dengue, nine cases of dengue with warning signs, and seven cases of severe dengue.

Severe dengue was characterized by hematemesis in six cases, renal failure in four cases, hepatic cytolysis in four cases, hematuria in two cases, and hemolysis and hepatic encephalopathy with altered consciousness in one case.

In cases of dengue accompanied by warning signs, the primary symptoms were persistent vomiting (n = 7), epistaxis (n = 3), and abdominal and epigastric pain (n = 2). One case of hemoglobinuria was also observed.

Table II shows that the clinical presentation of suspected dengue cases (n = 30) was mainly marked by fever (n = 25), headache (n = 22), and rash (n = 12).

**Table II:** Clinical characteristics of dengue suspected cases during the outbreak in the Savanna region of Togo in 2023 (n = 30)

**Clinical signs**	**headcount (n)**
Fever	25
Headache	22
Skin rashes	12
Muscle pain	9
Intense fatigue	7
Vomiting	6
Cough	6
Anorexia	5
Muscle soreness	3
Conjunctivitis, abdominal cramps, pruritus (*)	2
Diarrhea, runny nose, earache (**)	**1**

(*) 2 cases of each sign;

(**) 2 case of each sign

Hematological parameters showed an increase in white blood cell count in severe dengue cases. Biochemically, renal (urea and creatinine) and hepatic (alanine aminotransferase and aspartate aminotransferase) parameters increased in cases of dengue with warning signs and severe dengue (Table III).

**Table III:** Hematological and biochemical data of patients with dengue fever during the outbreak in the Savanna region of Togo in 2023 (n = 27)

	Classic dengue (n = 11)	Dengue with warning signs (n = 9)	Severe dengue (n = 7)
Hematology			
White blood cells (cells/mm^3^)	4 403,28 [2 000-10 000]	7 994,66 [1 800-14 920]	36 080 [15 900-59 900]
Platelets (cells/mm3)	188 090,90 [140 000-308 000]	115 444,44 [75 000-205 000]	84 142,85 [48 000-109 000]
Hemoglobin level (g/dL)	10,82 [9,7-13]	11,72 [6,2-15,2]	9,9 [8-12]
Biochemistry			
Uremia (g/L)	0,25 [0,1-0,45]	0,496 [0,21-0,52]	1,6 [1-2,04]
Creatinine (mg/L)	10,42 [7-14]	14,86 [8-19]	85,6 [36-125]
ALT (IU/L)	48,14 [30-74]	166,33 [49-302]	1 030,2 [366-1 895]
AST (IU/L)	70,14 [37-109]	236,8 [94-498]	1 143 [328-1 825]

ALT: alanine aminotransferase; AST: aspartate aminotransferase

The NS1 antigen of the virus was detected in 12 cases overall. IgM and IgG immunoglobulins were positive in 26 and 17 cases, respectively. Table IV presents the detection of the NS1 antigen and immunoglobulins (IgM and IgG) according to the clinical presentation of dengue (Table IV).

**Table IV:** Distribution of detection of NS1 antigen, immunoglobulins M and G according to the clinical form of dengue in patients during the outbreak in the Savanna region of Togo in 2023 (n = 27)

	Classic dengue (n = 11)	Dengue with warning signs (n = 9)	Severe dengue (n = 7)
	Positive	Negative	Positive	Negative	Positive	Negative
NS1 antigen	3	8	5	4	4	3
IgM	11	0	8	1	7	0
IgG	7	4	4	5	6	1

#### Therapeutic data

Symptomatic treatment was administered to all patients in various forms. Pain and fever were managed with paracetamol (n = 25) and metamizole (n = 20). Twelve patients received antiemetic treatment with metopimazine. Twelve patients received vitamin C infusions, and seven patients were prescribed hemostatics (etamsylate). Six patients required transfusions of packed red blood cells and platelet concentrate.

#### Progression data

In one patient with severe hemorrhagic dengue, progression was marked by:

- hepatorenal syndrome, consisting of hepatic cytolysis and hemolysis with jaundice, acute renal failure with oliguria, and portourine (hemolysis).
- respiratory distress with desaturation under non-invasive ventilation (oxygen saturation at 86%);
- sepsis.

Secondly, his condition improved after he was transferred to the Center for the Treatment of Diseases with Epidemic Potential in Lomé. There were two deaths, representing a case fatality rate of just over 7%. The first death was a 25-year-old patient with no underlying health conditions who presented with gastrointestinal bleeding, multiple organ failure (renal failure and hepatic cytolysis), and severe sepsis. The second death was a 65-year-old female patient with no history of disease who presented with renal failure, hepatic cytolysis, and hepatic encephalopathy.

The average length of hospital stay was 4.44 days, ranging from one to eight days.

## Discussion

Togo, a country bordering Burkina Faso that frequently experiences dengue epidemics, recently experienced its first major epidemic in the Savanes region. However, studies have already shown the circulation of the dengue virus in other regions of the country, such as Kara [24] and Grand Lomé [17]. Surveillance data enabled the collection of 57 suspected cases, including 27 confirmed cases and two deaths, representing a case fatality rate of 7%. These figures would be higher if reporting were effective and diagnostic tests were available. Although the disease is present in Togo, the country is at real risk of dengue due to its proximity to Burkina Faso; however, preparedness and response to a possible epidemic have not kept pace. At the start of the epidemic, the lack of locally available rapid tests meant that samples from suspected cases had to be sent to the national public health laboratory (Institut National d’Hygiène) in Lomé, which is 600 km from the Savanes region. The difficulty of accessing rapid dengue diagnostic test kits at the laboratory represents an initial recruitment bias. The list would have been longer if diagnosis had been possible on-site at the start of the epidemic. The risk of a dengue epidemic remains high in Togo because the disease is emerging and linked to the proliferation of the *Aedes* vector, which is caused by an increase in larval breeding sites. Vector control programs are failing, and climate change is a contributing factor [22]. The proliferation of the vector is due to overflowing cesspools in neighborhoods, defective or nonexistent drainage systems that lead to stagnant water, and terrain that is conducive to mosquito breeding. Uncontrolled urbanization and gaps in health policies to combat mosquitoes also contribute to this proliferation [8]. The country’s low socioeconomic status makes populations vulnerable to these diseases due to a lack of access to clean water and inadequate wastewater management, which promotes mosquito development and proliferation. Deforestation, certain agricultural practices, and the uncontrolled use of insecticides are additional factors that promote pathogen transmission by vectors.

*Aedes aegypti* is highly anthropophilic. Due to their high degree of domestication, the females lay their eggs in artificial breeding sites, such as water tanks, flower pots, used tires, and gutters. It has also been shown that the eggs can withstand desiccation for at least one year [5].

In 2016, the WHO gathered information from various studies and surveys in the African region and observed an increase of the *Ae. aegypti* vector throughout Africa, including certain North African countries [6]. This mosquito also carries other viral diseases, such as yellow fever, Zika virus fever, and chikungunya virus disease. The risk of a major epidemic through disease transmission is real and poses a threat to the continent and its populations’ health. Non-native disease vectors, such as *Ae albopictus* (the tiger mosquito), have been introduced to certain African regions [6], particularly West Africa (Nigeria and Benin), Central Africa, and East Africa (Gabon and the Seychelles Islands) [3,23].

The resurgence of insect vectors of infectious diseases is attributed, in part, to increased international trade and tourism. However, it is primarily attributed to climate change. According to some global studies, climate change is expanding the areas where the climate is more conducive to the proliferation of these insects and the associated epidemics [6]. The IPCC’s 1.5°C report on climate and health, published in October 2019, clearly states: “There is growing evidence that weather variations associated with climate change are altering the geographic range, seasonality, and intensity of infectious disease transmission”. In other words, *Aedes* vectors could spread well beyond their current range by 2030, as could the *Anopheles* mosquito, which carries malaria. An outbreak of this deadly disease has recently been noted in many African countries [6].

The current geographical distribution of the tiger mosquito, *Ae. albopictus,* encompasses regions with a climate similar to that of Chiba, Japan, the mosquito’s place of origin [6].

The epidemic occurred when several neighboring countries, including Burkina Faso, Ghana, Benin, Cote d’Ivoire, and Mali, were experiencing epidemics as well [16].

Burkina Faso borders the Savanes region of Togo. Since the first dengue epidemic in Burkina Faso in 1925, several others have occurred, including in 2013, 2016, and 2017 [10,14].

As of December 19, 2023, a total of 171,991 cases of dengue fever, including 70,223 confirmed and probable cases and 753 deaths (case fatality rate: 0.4%), have been reported in Burkina Faso, Cape Verde, Chad, Cote d’Ivoire, Ethiopia, Guinea, Mali, Mauritius, Sao Tomé and Príncipe, Senegal, Nigeria, Ghana, Benin, and Togo [19]. Burkina Faso accounted for 85% of total cases (n = 146,878) and 91% of deaths (n = 688) [16].

### Sociodemographic characteristics

The trend observed during the epidemic that showed dengue affected a young population has been noted in several West African epidemics, such as those in Burkina Faso [1,4,7,14].

### Clinical data

Several studies on dengue report a predominance of classic dengue cases with flu-like symptoms, primarily pain, as seen in our study, including headaches, myalgia, abdominal pain, retro-orbital pain, and neck pain [4,11].

In our series, the hemorrhagic syndrome was mainly characterized by hematemesis, epistaxis, melena, and hematuria. In Burkina Faso, from 2013 to 2017, cases of dengue were reported with symptoms of gingival bleeding, hematuria, hematemesis, metrorrhagia, and epistaxis [4]. In Senegal, during an epidemic in 2009, epistaxis and melena were the most common symptoms [7].

### Paraclinical data

Analysis of the hematological and biochemical data shows that the condition shifts from leukopenia (classic dengue) to hyperleukocytosis (severe dengue) as it progresses.

We also observed an increase in transaminases associated with dengue, especially in the AST fraction. A study in Singapore found that, out of 690 patients with confirmed dengue, 595 (86%) had elevated AST levels, regardless of clinical presentation. The more severe the dengue, the higher the AST levels [9]. This is fully consistent with the WHO classification criteria [20].

Due to the risk posed by dengue in Togo, the country has implemented several measures to strengthen border surveillance. Cross-border surveillance of dengue has been intensified through several actions, including briefing health workers at entry points, raising awareness among travelers and local populations about health risks, training health workers in high-risk areas to detect and manage cases early, and acquiring and making available the necessary supplies for treatment. An action plan has been put in place at the national level, encompassing the organization of case management in health facilities, strengthening early detection through laboratory confirmation, and raising public awareness of preventive measures against dengue [8].

## Conclusion

This study reports on the epidemiological characteristics of the first major dengue epidemic in Togo’s northern region, which borders Burkina Faso—a country where dengue is endemic and epidemics are frequent. A significant proportion of dengue cases presented warning signs. However, the virus serotypes could not be identified. Further studies are needed to determine the circulating serotypes, particularly those associated with severe dengue cases, in this region of Togo and throughout the country.

Due to its proximity to Burkina Faso, where the virus is more active, the Savanes region is a high-risk area for dengue fever, as is the rest of Togo. It appears that the North Togo-Burkina Faso region has become endemic for dengue fever. This finding should prompt the implementation of coordinated epidemiological surveillance of arboviruses in the region to determine their incidence and identify the viruses involved, as well as distinguish them from malaria.

## Funding

This study did not receive any funding.

## Authors’ contributions

Kpegba KA, Hilim M., and Kozon D. regularly supervised the updating of the linear list of dengue cases in the Savanes region.

Bawe LD, Kotosso A, and Abaltou B drafted the study protocol.

Bawe, Kotosso, and Abaltou analyzed the results and drafted the first version of the manuscript. Patassi AA and Watéba MI validated the results and corrected the first version of the manuscript.

## Conflict of interest

The authors declare no conflict of interest.
